# Early Neonatal Pain—A Review of Clinical and Experimental Implications on Painful Conditions Later in Life

**DOI:** 10.3389/fped.2020.00030

**Published:** 2020-02-07

**Authors:** Morika D. Williams, B. Duncan X. Lascelles

**Affiliations:** ^1^Department of Clinical Sciences, College of Veterinary Medicine, North Carolina State University, Raleigh, NC, United States; ^2^Translational Research in Pain Program, North Carolina State University, Raleigh, NC, United States; ^3^Comparative Medicine Institute, North Carolina State University, Raleigh, NC, United States; ^4^Thurston Arthritis Research Center, University of North Carolina at Chapel Hill, Chapel Hill, NC, United States; ^5^Center for Translational Pain Medicine, Duke University, Durham, NC, United States

**Keywords:** neonatal pain, early life, consequences, neurobiology, mechanisms, animal models, chronic pain

## Abstract

Modern health care has brought our society innumerable benefits but has also introduced the experience of pain very early in life. For example, it is now routine care for newborns to receive various injections or have blood drawn within 24 h of life. For infants who are sick or premature, the pain experiences inherent in the required medical care are frequent and often severe, with neonates requiring intensive care admission encountering approximately fourteen painful procedures daily in the hospital. Given that much of the world has seen a steady increase in preterm births for the last several decades, an ever-growing number of babies experience multiple painful events before even leaving the hospital. These noxious events occur during a critical period of neurodevelopment when the nervous system is very vulnerable due to immaturity and neuroplasticity. Here, we provide a narrative review of the literature pertaining to the idea that early life pain has significant long-term effects on neurosensory, cognition, behavior, pain processing, and health outcomes that persist into childhood and even adulthood. We refer to clinical and pre-clinical studies investigating how early life pain impacts acute pain later in life, focusing on animal model correlates that have been used to better understand this relationship. Current knowledge around the proposed underlying mechanisms responsible for the long-lasting consequences of neonatal pain, its neurobiological and behavioral effects, and its influence on later pain states are discussed. We conclude by highlighting that another important consequence of early life pain may be the impact it has on later chronic pain states—an area of research that has received little attention.

## Introduction

Newborn babies inevitably experience pain as a part of routine neonatal care, such as vitamin K injections and heel sticks to obtain blood for screening tests. These painful experiences are more frequent in infants who are sick or premature. In recent decades, much of the world has experienced a continued increase in preterm births with an estimated 15 million babies born prematurely each year globally (1). Preterm birth is defined as a baby born before 37 weeks of gestation and is further sub-categorized based on gestation age: extremely preterm (<28 weeks), very preterm (28– <32 weeks), and moderate to late preterm (32– <37 weeks) (2). A 2010 study of 184 countries found that the preterm birth rate, expressed as a percentage of livebirths, ranged between 5 and 18% and that this rate did not correlate directly to the economic development level of the country, e.g., northern European countries have a 5% rate whereas the United States is at 13% (3, 4). In the Unites States alone, the medical care, loss of productivity, maternal delivery, early intervention, and special education services are estimated to have cost $26.2 billion in 2005 (5). Furthermore, prematurity is the leading cause of death in children under the age of 5 years (1). Together, these factors make prematurity a modern global health crisis.

Though preterm births continue to increase, medical advancements in neonatal medicine have led to improved survival rates for preterm babies. This increase is associated with intense medical and surgical management over an extended hospital stay. Babies born at term (39–41 weeks) spend 4.9 days in the neonatal intensive care unit (NICU) (6). However, when adjusted for risk (i.e., birth weight, sex, small for gestational age status, ethnicity, fetal distress, and maternal stress), the average length of stay in the NICU for extremely preterm infants is 81 days (7). During this period, newborns may undergo multiple painful procedures each day for care and treatment. The demographics of the NICU is predominately premature newborns (24–36 weeks) when compared to term newborns (37–42 weeks)-−72.3 and 27.7% of NICU babies, respectively (8). Therefore, although preterm babies are at a higher risk for neonatal pain exposure, term newborns are not exempt. In fact, in the United States, ~460,000 neonates require NICU admission each year, and thus experience procedural, medical, or surgical pain (9).

The neonatal nervous system is very plastic because it is immature and undergoing major developmental changes; therefore, neonates may be vulnerable to neurodevelopmental changes from painful stimuli (10). Nociceptive input during early neurodevelopment is typically deleterious, increasing the risk of neurodevelopmental impairments, including short- and long-term physical and psychological disability (4, 11, 12), adverse changes in brain development and processing (13–15), and alterations in somatosensory function that lead to pain (16, 17). Such damage may have significant long-term effects that persist into childhood and even adulthood; a notion increasingly supported by the literature (17, 18).

The purpose of this report is to describe the current understanding of the relationship between early life pain and its implications on pain later in life, via a narrative review of clinical and pre-clinical studies. To this end, we highlight common painful procedures that occur early in life and the important consequences of these pain events to humans later in life. In each section we discuss the preclinical work that has advanced our understanding of these consequences and the potential mechanisms underlying them. We then review the animal model correlates used to investigate the effects of early life injury and discuss future directions for research.

## Methods

The literature review was performed by searching the following databases, including PubMed, CAB Abstracts, and Google Scholar. Search was conducted using the following keywords: early life injury, early life pain, neonatal injury, neonatal pain, repetitive needle prick, heelstick, consequences, impairment, behavior, cognition, pain response, central nervous system, mechanisms, pain models, animal models, chronic pain, osteoarthritis, and inflammatory pain. For articles generated from this search, the abstracts were read, and then those relevant were downloaded and read in full. Those fitting in topic of relevance were human studies or rodent models of neonatal pain. Rat studies and behavioral studies were emphasized for the review. Studies investigating the effects of stress and other environmental factors were excluded from the review.

## Painful Events Occurring During Early Life in Neonates

Newborns routinely experience invasive, painful procedures, such as blood collection, immunization, vitamin K injections, and/or circumcision. Preterm or sick term newborns in the NICU experience a much higher frequency of invasive and painful procedures (8, 18). On average, premature babies requiring NICU admission encounter about 14 painful procedures daily in the hospital (19, 20). These invasive procedures range from repetitive heel sticks to minimally invasive or open surgery (Table 1) (21–24).

**Table 1:** Painful procedures commonly performed in the NICU.

*Diagnostic* Heel stick Venipuncture Arterial puncture Bronchoscopy Endoscopy Lumbar puncture Retinopathy of prematurity exam	*Therapeutic* Bladder catheterization Central line insertion/removal Chest physiotherapy Umbilical vessel catheterization Dressing change Gavage tube insertion Intramuscular injection Peripheral venous catheterization Mechanical ventilation Postural drainage Removal of adhesive tape Suture removal Tracheal intubation/extubation Tracheal suctioning Ventricular tap	*Surgical*Circumcision Cardiac surgery Congenital anomaly repairs Minimally invasive surgeries (laparoscopy, thoracoscopy)

*Information taken and adapted from Anand and International Evidence-Based Group for Neonatal (21) and Johnston et al. (22)*.

One of the most frequently performed invasive procedures in the NICU is a heel stick, or heel lance/needle prick (8, 25). This procedure is a pinprick puncture into the heel of a newborn used to obtain blood samples for screening laboratory tests, glucose levels, general chemistries, complete blood counts, and toxicology screening. Generally, to obtain adequate amounts of blood for testing, the heel must be squeezed, which is more painful for newborns than venipunctures (26). Despite this fact and the recommendation to use less invasive alternatives (e.g., venipuncture, mechanical lances) or to incorporate non-pharmacological analgesia to minimize the pain (9), heel sticks continue to be common in practice.

Another painful procedure that may be required for newborns with significant respiratory compromise is endotracheal intubation, a procedure performed on both adults and infants that allows access to the airway to provide respiratory support via mechanical ventilation. Adult patients report mechanical ventilation to be a source of pain, discomfort, and depression (27). Because the procedure is known to be associated with pain (28, 29), the International Evidence-Based Group for Neonatal Pain recommends this procedure be performed ‘without the use of analgesia or sedation … only for resuscitation in the delivery room or for life-threatening situations associated with the unavailability of intravenous access’ (22). However, clinical use of pain control is still suboptimal (30) despite evidence that opioids reduce signs of pain during mechanical ventilation in newborns (31). In addition, there is concern around the long-term negative effects of opioids, such as neuromotor disability (32, 33). In the United States alone, mechanical ventilation is provided to ~35,000 preterm and 20,000 term neonates each year (34). Therefore, there is great concern that mechanical ventilation is a significant contributing factor to the early life pain experience.

To manage and correct life-threatening diseases or abnormalities, open and/or minimally invasive surgeries may be required. Although the exact number of surgeries performed on newborns is unknown, up to 33% of extremely preterm babies require surgery to treat congenital anomalies or manage complications (17, 35). Multiple surgeries may be needed, thus increasing the exposure to other painful procedures, such as venipunctures, endotracheal intubations, and anesthetic episodes—as well as the surgical trauma-associated and post-operative pain itself. As the procedural numbers rise, the likelihood of developing hypersensitivity and/or persistent pain at the site of damage (chronic post-surgical pain) increases within months following surgery (18, 36, 37). Chronic post-surgical pain is thought to be in large part due to a phenomenon called central sensitization, a manifestation of maladaptive responses of the spinal cord that lead to increases in excitability and reductions in inhibition resulting in enhanced pain responsiveness (38, 39).

## Consequences of Early Life Pain in Humans and Experimental Models

Despite the high incidence of painful procedures performed in the NICU, most neonates receive no pharmacological analgesia (40–42) or receive an inadequate level of analgesic (43, 44). A combination of factors, such as the underestimation of pain or concerns for potential adverse effects, lead practitioners to withhold or inadequately dose these medications. However, recent studies exploring the long-term consequences of untreated, early life pain have shown that early life pain can have negative repercussions that can impact sensorimotor and cognitive development, behavior/mood, pain responses, pharmacological requirements, and health status seen as early as adolescence. We discuss these consequences below.

### Long-Term Neurosensory and Cognitive Impairments

It is believed that early pain experiences shape the somatosensory scaffolding of later perceptual, cognitive, and social development (45). This belief is evidenced by a recent study that found an association between painful procedures in early life and a reduction in brain responses to non-noxious touch stimuli (45). Such risks of neurosensory impairment appear to be aggravated by prematurity. Sensorimotor impairments in children, such as impaired vision/blindness, impaired hearing/deafness, cerebral palsy, delayed development trajectory, and impaired intellect into childhood and adulthood have been attributed to painful medical procedures early in life (46). For example, neonatal surgery has led to major neurosensory disability with effects that persistent into childhood (8 years old), with males having a higher risk of disability (47).

Early exposure to painful procedures can negatively impact neurodevelopment, such as brain growth, which is directly related to cognition. For example, an inverse correlation between frequency of invasive procedures and amygdala and thalamus volumes was seen in 8 years old children born very preterm (48). Further, reduced brain volumes in children who survived very preterm birth and experienced early life pain were associated with poor cognitive outcomes, such as lower intelligence (IQ), language and attention deficits, poor visual-motor functions, and poor behavioral outcomes (48–51). These cognitive deficits have been shown to persist into early adolescence and young adulthood (19 years old) for those who are born premature (52–54). Overall, preterm boys appear to be at a greater risk for poor neurodevelopmental trajectories than their female counterparts (53, 55). In contrast, females with early pain seemed to be more vulnerable to brain development with slower growth of thalamic, basal ganglia, and total brain volumes (56). However, conflicting sex-differences may be attributed to varied study designs, outcome measures, environment, observer variation, or individual variation.

Rodent studies have found similar impairments to cognition and brain development caused by early life pain. In a rat model of neonatal pain, mimicking NICU care, long-term alterations in brain development were seen (57, 58). Rats exposed to acute repetitive needle prick stimulation (4 times per day for the first 2 weeks of life) suffered long-term memory impairment (59), while chronic repetitive needle prick stimulation (4 times per day, every other day, for the first 8 weeks of life) resulted in short term memory deficits (60). Similarly, neonatal rat inflammatory pain produced long-lasting alterations in hippocampal function that were more pronounced in middle-aged adult (P424–442) vs. adult (P144–158) rats, characterized by spatial memory impairment (61). Ranger et al. report that memory in adulthood was poorer for mice exposed to repetitive pain during the first week of life (62).

### Negative Impacts on Psychosocial Behaviors

There is a strong association between exposure to painful procedures and altered behavioral development trajectories (63). Survivors of early repetitive pain may develop attention-deficit disorders, atypical behaviors, such as hypervigilance and exaggerated startled responses, and other forms of long-term stress-related psychosocial disabilities (64). Neonatal rodent models support these findings by showing that repetitive acute pain during the first week of life can lead to negatively altered locomotor activity (65), defensed withdrawal behaviors (57), anxiety/depressive behaviors (66), and reduced social behavior (67).

Internalizing behaviors are negative actions that are directed toward oneself manifesting as withdrawal, depression, and/or anxiety (68). Higher levels of internalizing behaviors are predictive of atypical social competence in children leading to greater social difficulties and lower peer acceptance (68, 69). These negative behaviors have been seen in children as early as 18 months old (70) and may persist throughout childhood and adulthood (54, 71, 72). Preterm children exposed to neonatal pain have a higher rate of internalizing behaviors than full term children (70, 73). For example, young adults born extremely preterm who underwent prior surgery had higher anxiety and pain catastrophizing than term controls (74). Internalized behaviors can also lead to the development of other serious health issues, such as drug addiction (75), alcoholism (76), or obesity (77).

### Varied Behavioral Pain Responses

Early life pain can be challenging to recognize due to variability in patients' responses toward pain and changes associated with age and subsequent painful experiences. Changes in facial expression and withdrawal reflex in the neonate are suggested to be most associated with nociception activity (78). However, facial grimacing after a heel stick was seen as early as 28 gestational weeks of age, becoming more recognizable with age. Recently, Green et al. demonstrate the emergence of discriminative facial expressions toward noxious and innocuous stimulation occurring in infants at ~33 weeks' gestation, coinciding with brain maturation (79). Some work has shown that the degree of grimacing was inversely related the number of invasive procedures (43). Additionally, a greater number of procedures during early life has been associated with lower behavioral pain responses (e.g., facial grimace, cry, state of arousal) and pain scores following subsequent pain exposure within 1 month of life (80–82). Although pain experiences may not produce noticeable behavioral changes, noxious stimulation (i.e., heel stick) in infants between 25 and 43 gestational weeks may be processed at the somatosensory cortical level (83). The presences of cortical activation, measured by increases in hemodynamic responses, without a facial motor response support the concept that pain is reflective of emotional responses requiring cortical processing (83, 84). Sex should also be considered when evaluating facial grimacing, as female preterm neonates showed more robust facial expressions than males in response to acute procedural pain (85).

### Reorganization of Pain Processing

Painful procedures during neonatal development have been shown to have later influences on sensitivity to noxious stimuli and pain. Negative hemodynamic effects, such as higher heart rates and lower oxygen saturations, following secondary noxious stimuli are reported in individuals with a history of neonatal pain (43, 80, 86). Males with a history of neonatal pain have more pronounced responses than females with respect to hemoglobin oxygenation following secondary venipuncture (87).

Procedural pain early in life appears to lead to alterations in neonatal sensory function resulting in hyposensitivity following acute pain as a neonate but hypersensitivity later in life (88). However, the directionality of changes in sensitivity later in life is inconsistent. Preterm or full term children between 10 and 12 years old who underwent neonatal surgery have shown generalized thermal hyposensitivity as well as mechanical and thermal hyposensitivity in areas around prior tissue damage (89, 90). In contrast, in preterm adolescents between 12 and 18 years old there was greater mechanical hypersensitivity compared to full term children, with females being consistently more sensitive than male counterparts (91). And, young adults (18–20 years old) who were extremely preterm and underwent prior neonatal surgery had higher pain intensity scores and reported moderate to severe persistent pain more than young adults who were term born (17, 74).

Investigations into whether painful early life experiences have a negative impact on pain responses following secondary acute injury later in life have also been conducted. Hypersensitivity and allodynia after secondary injuries have been demonstrated (88). Heightened pain responses (e.g., cried longer, higher pain scores) toward routine vaccinations given to 4–6 months old neonatally circumcised babies were seen when compared to uncircumcised babies (92, 93). Infants (4–21 weeks of age) requiring repeated surgery in areas of scaring, due to prior neonatal surgery, had higher post-operative pain scores and higher analgesic requirements than controls (94). Furthermore, higher pain intensity ratings during venipuncture at age 7.5 years were positively associated with a greater number of invasive procedures experienced in the neonatal period (95). Variations in pain responses (e.g., becoming hypersensitive or hyposensitive) with or without secondary injury are likely due to difficulties with controlling for: intensity/frequency of pain stimulus, time/age following painful event(s), and variability within study population, outcome measures, and other environmental influences.

Animal models of pain have aided in further exploration of these effects of early life pain as well as provided additional support for these clinical findings. After repetitive needle prick stimuli, neonatal rats had decreased thermal sensitivity during development (57) and adulthood (60). Similarly, adolescent rats demonstrated peripheral thermal hypoalgesia after experiencing repeated post-natal inflammatory peripheral pain (formalin) (96). In contrast, repeated neonatal hindpaw incision or needle prick has been shown to sensitize adult rats to secondary incisional injury resulting in increased incision-related hyperalgesia to cutaneous dynamic tactile (brush), pinch, and/or punctate (von Frey hair) stimuli (16, 97–100). For additional results, Walker et al. provides a list of long-term effects of neonatal injury demonstrated in laboratory models (101).

### Increased Poor Health Outcomes

Early-life, pain-induced alterations can increase the likelihood of having subsequent poor health outcomes. Preterm babies, who are exposed to painful stimuli, are at a greater risk of developing non-communicable diseases (diabetes, hypertension) and other health conditions later in life (102), creating an intergenerational cycle of risk. Controlling for confounding factors, post-natal growth is reduced in infants that undergo repetitive procedural pain during early life (103), and infants who are smaller tend to have more cognitive problems than other children as previously discussed. Small infants often rapidly catch-up in growth during the first 2 years of life, but this ‘catch up’ is associated with the development of adiposity and impaired insulin sensitivity later in life (104). The development of these outcomes may lead to increased risk of obesity and metabolic disorders, such as type II diabetes in humans (105, 106) and in animals (107). Blood pressure in adults born premature may be higher than adults born full term, and thus prematurity exposes one to a higher risk of hypertension, cardiovascular disease, and stroke (108). People with these health conditions are predisposed to becoming chronic pain sufferers. There is a growing body of literature regarding the long-term health effects of early life injury, and a detailed, more exhaustive list can be found elsewhere (15, 109). Additionally, more clinical and research studies have been conducted to investigate the persistent ramifications of early life pain beyond infancy (67, 110–112).

## Neurobiological Mechanisms Underlying Enhanced Pain Responses to Subsequent Injury

Pain is a complex, multidimensional, and multisensory phenomenon involving many intact systems to produce the final emotional and sensory response. For the perception of pain, a noxious stimulus must be processed through the various levels of the neuroaxis, and arrive at higher brain centers. Nociception, or pain responses, can be inhibited at the level of the spinal cord through the descending inhibitory pain pathway. The descending pain circuit is composed of the periaqueductal gray matter in the upper brain stem, the locus coeruleus, the nucleus raphe magnus, and the nucleus reticularis gigantocellularis. Under normal conditions, the descending pain modulatory system controls the balance between facilitation and inhibition of nociception, maintaining a baseline of sensory processing (113). However, disruption in the system can lead to enhanced or facilitated nociception or promotion and maintenance of chronic pain (114).

The pain transmission system is further complicated by interactions between the central and peripheral nervous systems and the immune system—neuroimmune interactions. Neuroimmune interactions are responsible for: recruiting local neuronal elements for fine tuning the immune response; participating in the plasticity of synapses during development as well as in synaptic plasticity at adulthood; and engaging the rest of the body in the fight against infection from pathogenic microorganisms (115). This established bidirectional interaction between the neuronal and immune systems plays a crucial role in pain modulation (116–118).

During neonatal development newborns have a high degree of neuroplasticity and are very vulnerable to the modulating effects of noxious stimuli (88, 119–121), especially if repetitive in nature. Repetitive painful stimuli cause structural and functional reorganization of the nervous system at the level of the: periphery, spinal cord, and supraspinal pain processing; neuroendocrine function; and neurologic development (64, 82, 122). Human and animal studies have provided solid evidence that repetitive and prolonged pain early in life can alter subsequent pain processing (43, 123) and later pain sensitivity (59, 124). It remains to be determined if such changes also contribute to chronic pain. Specific underlying structural and functional reorganization that may be responsible for enhanced pain responses to subsequent pain as a result of early life pain are discussed here, with an emphasis on information gained from preclinical work in animal models.

### Peripheral Nociceptive Fiber Development

It was once believed that neonates were unable to perceive pain due to the immaturity of the sensory nervous system (125). However, neuroanatomical and behavioral studies have provided strong evidence to the contrary (126–128). During the neonatal period, a considerable amount of maturation associated with pain transmission and modulation is taking place. Although the peripheral nervous system is mature and functional by 24 gestational weeks, there are substantial neuroanatomical shifts in the distribution of unmyelinated (C) and myelinated (A) ascending fibers occurring in the post-natal period (41). Early in life there is an abundance of myelinated Aδ sensory fibers, responsible for the initial perception of pain, and fewer low density C fibers, responsible for pain intensity (129). Pre-clinical non-human animal studies have further demonstrated the relative numbers of fiber types can be affected by early life pain, leading to hyperinnervation and subsequent peripheral sensitization and increased pain sensitivity later in life (130). Babies are more likely to have hypersensitivity due to imbalances between the numbers of afferent fibers and descending (negative) influences (124).

### Spinal Cord Mechanisms

The spinal cord can undergo neuronal and synaptic changes due to inputs from the periphery, known as activity-dependent plasticity (131, 132). Spinal cord plasticity shapes its function during post-natal development and is continuously occurring throughout life (131, 133). Peripheral C-fiber activity drives activity-dependent cellular wind up, which initiates widespread changes in the function of the neuronal network in the spinal cord; this leads to clinical manifestations of spontaneous pain, abnormal sensitivity to noxious stimuli, or even innocuous stimuli, and referred pain that often follows injury to peripheral tissues (132). Changes induced by peripheral input depend on the intensity, duration, and the life stage at which the input occurs. Animal model research has been crucial to advance our understanding of the neuroanatomical and functional changes, and the effects on sensitivity to subsequent stimuli, produced by early life noxious stimuli. Neonatal rats exposed to a peripheral inflammatory stimulus in the hindpaw appear to have enhanced responsiveness to sensory stimulation due to altered axonal patterns in the substantia gelatinosa; these functional changes suggest that axons are sprouting into new areas of the dorsal horn (134). Furthermore, a single noxious input (CAR) at different ages between P0 and 8 in the neonatal rat has led to sustained thermal and mechanical hyperalgesia after re-inflammation (CFA at P40) in the ipsilateral, but not contralateral, hindpaw that is likely a result of local segmental circuitry involvement (135).

At the level of the spinal cord in rats, the balance between excitatory and inhibitory neurotransmitters shifts with maturity. In adults, substance P, calcitonin gene-related peptide, and glutamate are the neurotransmitters involved in excitatory pain transmission; γ-aminobutyric acid (GABA), norepinephrine, glycine, adenosine, endogenous cannabinoids, and opioid peptides are involved with inhibitory pain modulation (136). During early neurodevelopment (e.g., first post-natal week of rat life), GABA has an excitatory effect in the hippocampus caused by an inverted chloride gradient that induces depolarization (137). Similarly, there is a transient GABAergic excitatory effect in the immature dorsal horn; however, this shifts to inhibitory signaling by the end of the first post-natal week (138, 139).

Bremner et al. suggests that spinal GABAergic inhibitory transmission onto single dorsal horn cells “*in vivo*” is functional at P3 in rats and low chloride extrusion capacity does not impede the normal inhibitory functions of GABA (140). GABA activates voltage-gated sodium and calcium channels and potentiates the activity of N-methyl-D-aspartate (NMDA) receptors by reducing their voltage-dependent Mg^2+^ block (137). NMDA receptors, thought to be responsible for central sensitization (141), and activity-dependent tuning expand receptive fields in the dorsal horn until 42 gestational weeks, gradually reducing to adult size by 44 gestational weeks in humans (41). Together, increased expression of NMDA receptors and shifts in GABA function creates an excitatory neuronal environment during the most vulnerable period of a baby's life, when they may be exposed to multiple noxious insults. In an excitable neuronal environment, noxious and non-noxious stimuli may result in hypersensitivity, which is exacerbated with repeated stimuli (41).

### Descending Modulatory Systems

In the mature nervous system, descending pathways are responsible for inhibiting noxious signals at the level of the spinal cord. Animal research has been crucial in understanding the maturation of descending modulatory systems, and therefore when the neonate is most vulnerable. During infancy the descending modulatory system is facilitatory, which is mediated by the mu-opioid receptor pathways in the rostroventral medulla (RVM) in the rat (142). As the RVM matures (e.g., P25–P40 in rat), the control over spinal nociceptive circuits transitions to descending inhibition (142). However, prior to P40, without full development of the descending inhibitory mechanisms, endogenously driven suppression of noxious stimuli from the periphery is not fully functional, thus the neonatal nervous system is more vulnerable to the effects of noxious stimuli (120, 121, 128, 143). Early noxious experiences can lead to permanent or long-lasting changes of the RVM circuits and other inhibitory mechanisms.

The vulnerability of the neonate to long term changes induced by noxious stimuli prior to maturation of the descending inhibitory pathway is highlighted by animal research. Neonatal incisional injury (P3) modifies descending pathways of spinal excitability from the RVM in early adulthood, producing acute hyperalgesia but long-term generalized hypoalgesia (P28) (144). In contrast, repetitive noxious stimulation (needle prick) in the neonatal rat (P0–14) has led to long-term thermal hypersensitivity (P56), likely due to alterations in the descending modulatory circuitry (59). Additionally, neonatal tissue inflammation (carrageenan; P3) has shown to lengthen thermal withdrawal latencies at the site of injury compared to controls; however, re-inflammation later in life (CFA; P50) exaggerates nocifensive responses, such as peripheral thermal hyperalgesia (145). These alterations in behavioral responses post-CFA re-injury are likely a result of enhanced RVM-evoked modulation of the pain reflexes, thus strengthened descending inhibition of the spinal cord (145).

### Brain

Pruning, or the selection of active neuronal circuits, occurs throughout life; however, during the late second trimester and throughout the neonatal period, these processes and others that shape the neural architecture are particularly active in the human brain (146). In the neonatal period, there is rapid neuronal proliferation and differentiation including: maturation of oligodendrocytes; distribution and activation of microglia; differentiation, migration, and proliferation of cortical neurons; development of the subplate neurons, cerebral cortex, deep nuclear structures, and axons; formation of synaptic connections; and increase in cortical surface area and gyral formation (147). As discussed above, procedural pain in the NICU can have dramatic effects on brain development and function (13, 56).

Neonatal pain has been associated with reduced brain microstructure and volumes in humans (56) and pigs (148). Proposed mechanisms responsible for reduced brain volume and structure due to early life pain include alterations mediated by excitotoxicity and disrupted axonal development. Persistent neonatal pain in the rat can result in neuronal death in cortical and subcortical areas due to excitoxicity (149). Additionally, microglial activation in the rat initiates downstream intracellular cascades (e.g., release of glutamate, proinflammatory cytokines) that can result in cell death via excitotoxicity (150). Human imaging studies (e.g., diffusion tensor imaging, MRI) suggest pain-associated impairment in axonal development as another mechanism responsible for abnormal brain development (13). For example, early procedural pain in the premature newborn likely contributed to the development of damaged subcortical neurons with secondary axonal changes in the white matter (13).

Regions of the brain that appear to be most affected by early life pain and noxious input are associated with or connected to the limbic system (e.g., hippocampus, amygdala, and thalamus) and basal ganglia (48). In studies conducted by Nuseir et al., lower levels of hippocampal neurotrophins were detected in injured neonatal rats as compared to control rats, and the authors suggested this as the underlying mechanism for memory impairment (59, 60). The thalamus is the relay center for sensory and motor signals ascending to the cerebral cortex. Human infants who experience pain have decreased thalamic volumes, accompanied by disruptions in thalamic metabolic growth and thalamocortical pathway maturation (14). Thalamocortical connections undergo rapid formation during gestation and early post-natal life making them particularly vulnerable to excitotoxicity (151). Interestingly, the rat thalamus is regulated by NMDA-dependent long-term potentiation, which is only active during the first week of life (152), when newborns are routinely exposed to painful procedures. Therefore, early pain can alter thalamic development due to NMDA-dependent mechanisms. Furthermore, NMDA and GABA receptors are also key components of synaptic plasticity in the amygdala of the rodent and are presumed responsible for neuronal responsiveness and structural reorganization (153). Early life insults in the piglet can alter the normal developmental expression of GABA receptors, thereby impacting neuronal cytoskeleton development and myelination (154), which may lead to downstream effects on learning and cognitive impairments. These changes within the limbic system appear to be conserved across species throughout life affecting lifelong memory and associated fear (153, 155, 156). Factors and mechanisms adversely influencing brain development are being elucidated by using animal models, but there is much more to understand about the cellular and molecular mechanisms.

### Neuroimmune Plasticity

The neonate's immune system is immature and undergoes rapid changes early in life (157). It is well-understood that the immune system plays a critical role in pain modulation due to the tightly woven interactions of the nervous system and immune system (116–118). Specific components of the immune system play an intimate role in modulating pain sensitivity (158). A key player in immune-related pain are microglia cells, which make up 15% of the total glial population in the central nervous system (CNS) (159). Microglia are present during the embryonic and early post-natal period during which they inhabit the spinal cord and brain (160). Animal research has shown that the development and architecture of the CNS is fine-tuned and shaped by microglia, where they also contribute to neuroplasticity and lifelong maintenance and protection of the CNS (161). Microglia respond to noxious stimulation by activating and proliferating, which contributes to maladaptive pain and central sensitization. Microglia reactivity is a complex multistage activation process, where microglia undergo a morphological and functional transformation process. Early life challenges, including tissue injury, lead to microglial activation that may result in the release of numerous signaling molecules that can have deleterious effects on the pathogenesis of pain, especially in the neonate (161, 162). For example, spinal microglial reactivity, measured by immunostaining of microglia-specific ionized calcium-binding adapter molecule 1 (Iba-1), was enhanced in rats exposed to a noxious insult (e.g., incision, inflammation) as a neonate (16, 88, 163–165).

With subsequent noxious experiences, activated microglia are primed to respond to injury more rapidly. Microglial priming appears to last for extended periods of time. As a result, microglia have gained attention as a suggested cellular mechanisms linking early life pain to altered pain processing and chronic pain in adulthood (161). Thus, microglia have become a major focus of investigative pre-clinical studies into pain processing pathways, neuroplasticity, and as potential treatment targets. However, the mechanisms driving microglial priming are unknown, and it is unknown how long microglial priming lasts. Interestingly, a murine study suggests that while male mice require microglial activation to develop pain hypersensitivity, females have an alternative route via the adaptive immune system (166).

## Models to Study the Effects of Early Life Pain on Pain Models to Study the Effects of Early Life Pain on Pain Occurring Later in Life

The most commonly used clinically relevant models (we use ‘model’ to refer only to the induced pain state) of neonatal early life pain are the repetitive needle prick (RNP) (37, 57) and plantar incision (16, 100, 144), most often using a rat. These painful insults are performed at various time points during the early post-natal period (P0–P7) in the rat because this critical period corresponds with that of a preterm human infant (24–36 weeks gestation) (167). The RNP model involves repeated tissue-breaking injury to the rodent's hindpaw using a needle to mimic the heel stick frequently performed in human babies in the NICU (8). Usually the noxious stimulus is administered between 4 and 8 times per day over a defined period of time during the early neonatal period (P1–14).

The plantar incision model involves incising the skin and fascia of the mid-plantar aspect of the hindpaw and the underlying plantar muscles followed by routine closure. The plantar incision is a clinically relevant model used to mimic surgical injury and has been used in animals (e.g., rats, pig) of varying ages to investigate post-operative incisional pain and the efficacy of various analgesic treatments (168–173). In the context of neonatal early life pain, the incisional model has been used in rats between P3 and P17 (97, 144). Both paradigms result in reliable neonatal tissue-breaking injury and nociceptive input thus serving as clinically relevant models to better understand the mechanisms and consequences of early life pain.

Other models used to evaluate the long-term effects of neonatal injury include the induction of inflammation (e.g., carrageenan, CFA), full-thickness skin wounds, laparotomy, intramuscular injections, and peripheral nerve injury models (Tables 2, 3) (101). Alternative models have focused on the psychological implications of early life pain and prematurity, such as stress (67). The most commonly used species are rodents; however, there are reports of other species being used. For example, piglets who undergo tail docking (P3 or P63) have long-term peripheral mechanical hyperalgesia (tail stump) (212) and distinctive changes in gene expression up to 16 weeks following the procedure (213).

**Table 2:** Pain models used to evaluate long-term effects of neonatal pain with or without subsequent pain later in life.

**1st Stimulus**	**2nd Stimulus**	**References**	
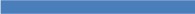		(165 , 174 , 175 )	
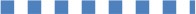		(134, 135, 165, 175–179)	
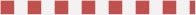		(58, 180)	
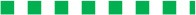		(165, 181–184)	
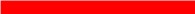		(57, 59, 62, 177, 184, 185)	
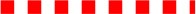		(97, 100, 178, 186–188)	
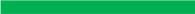		(189–192)	
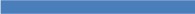	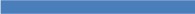	(193, 194)	
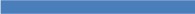	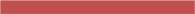	(195)	
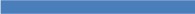	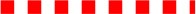	(171)	
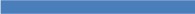	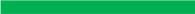	(194)	
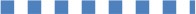	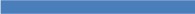	(135, 145, 196–198)	
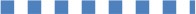	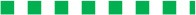	(199)	
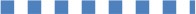	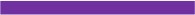	(61)	
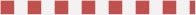	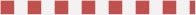	(200)	
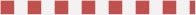	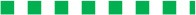	(96)	
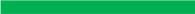	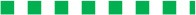	(201)	
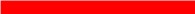	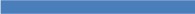	(202)	
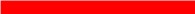	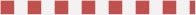	(203)	
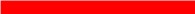	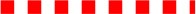	(37, 98, 204)	
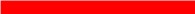	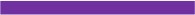	(205)	
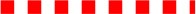	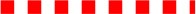	(16, 97, 100, 163, 206, 207)	
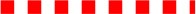	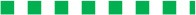	(144)	
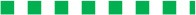	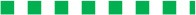	(164, 208, 209)	
*This illustration represents different pain models used to mimic injury that occurs early in life (P0–17) and/or later in life (P6–125). These models have been used to evaluate the long-term consequences of neonatal pain and inflammation*.			
*key*	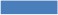 *Complete Freud's Adjuvant* (CFA)	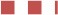 *Formalin*	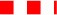 *Incision*
	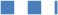 *Carrageenan (CAR)*	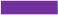 *Stress*	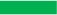 *Nerve Injury*
	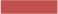 *Capsaicin (CAP)*	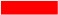 *RNP*	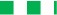 *Other (e.g. LPS)*

**Table 3:** Rodent models of neonatal pain and times of assessment.

**“1-Hit” Models**			
**1st Stimulus (age)**	**Outcome measure(s)**	**Age/Time**	**References**
**INFLAMMATORY PAIN**			
CFA (P1)	PWL EPM, PWL, sleep recordings, ACTH, corticosterone	P30 P90	(174)
CFA (P1)	PWT, PWL, paw diameter DRG [ATF3, CGRP, IB4]	2, 4, 24, 72 h, then 2 × weekly until 8 weeks 12, 24 h, 3, 7, and 14 days	(175)
CFA (P10)	Paw diameter, PWT SC [IBA1]	0, 1, 3, 24 h, 7 days 1, 24 h, 7 days	(165)
CAR (P10)	Paw diameter, PWT SC [IBA1]	0, 1, 3, 24 h, 7 days 1, 24 h, 7 days	(165)
CAR (P1)	PWT, PWL, paw diameter DRG [ATF3, CGRP, IB4]	2, 4, 24, 72 h, then 2 × weekly until 8 weeks 12, 24 h, 3, 7, and 14 days	(175)
CAR (P0, 1, 3, or 14)	Pain behavior PWL DRG [CGRP, IB4], electrophysiology	P0, 1, 3, or 14 P56–70 P56	(134)
CAR (P3 or 12)	Paw diameter EPM, FST	0, 0.5, 1, 3, 6, 14, 24, 32, 39, 45, 52, 60, 69 h P50–55	(176)
CAR (neonate; *not specified*)	Electrophysiology (PWT)	P19–23	(178)
CAR (P3)	PWT	P6, 13, 20, 27, 34, 41, 48, 55, 62	(135)
CAR (P1, 4)	Fear conditioning PWT, PWL	P24, 45, 66 P27, 48, 69	(177)
CAR (P0)	OFA FST, sucrose preference, stress-induced analgesia test with PWL	P65–70 P70–90	(179)
CAR (P0)	OFA FST, corticosterone	P60–80 P70–90	(210)
Formalin (P1–2)	Formalin test (nociceptive activity) EPM, FST, MWM	P25 P25–38	208)
Formalin (P1–3, 1–5, or 10–12)	Brain histology, WB	P4, 6, 13	(58)
LPS (P14)	Brain (c-Fos) EPM Fear conditioning PWT	2.5 h P38–42, 62–68 P60–72 P60–90	(181)
Zymoson (P10)	Paw diameter, PWT SC [IBA1]	0, 1, 3, 24 h, 7 days 1, 24 h, 7 days	(165)
**TISSUE-BREAKING PAIN**			
Skin Flap (neonate; *not specified*)	PWT, electrophysiology	P19–23 or 40–44	(178)
RNP (P0–7)	Weight gain PWL Alcohol preference, defensive withdrawal, social discrimination, air-puff startle tests	P8, 15, 21 P16, 22, 65 P20–30	(57)
RNP (P1–7)	Fear conditioning PWT, PWL	P24, 45, 66 P27, 48, 69	(177)
RNP (P0–14)	PWL Radial arm water maze Hippocampus [ELISA]	P28, 56 P56, 57 P70	(59)
RNP (P0–7)	PWT OFA MWM OFA, Corticosterone, ACTH, WB	P8, 15, 22, 43, 57, 85 P25, 87 P24–30, 87–93 P24, 45, 87	(187)
RNP (P0–7 or 0, 2, 4, 6)	OFA EPM, FST, Corticosterone	>P98 >P119	(185)
RNP (P1–6)	OFA, EPM, MWM, Rotarod, PWL, Sucrose preference	P60–85	(62)
RNP (P8–14)	EPM MWM	P30 P31–33, 34–36, 38	(184)
Incision (P3, 10, 17)	PWT, PWL, OFA, brain [c-Fos]	>P90	(97)
Incision (P7 or 28)	Electrophysiology (PWL)	0, 1 h	(188)
Incision (P3 or 40)	PWT EMG	0, 4, 24, 48, 72 h 24 h	(100)
Incision (P3)	PWT, PWL Electrophysiology	Weekly, P14–42 P40	(144)
Incision (P6)	Development assessment Wire hang test Rotarod OFA, EPM NOR	P6–21 P17, 18, 20 P17, 19, 21 P18 P19	(186)
Toe clipping (P7 or 17)	Developmental assessment OFA, EPM Balance beam Rotarod	P6–21 P56 P63–65 P70–72	(182)
Toe clipping (P3 or 7)	Developmental assessment Grip test, PWL Corticosterone	1, 3, 5, 12 h, daily for 3 weeks P84–86 P7	(183)
Tail clip (P8)	EPM MWM	P30 P31–33, 34–36, 38	(184)
**NERVE PAIN**			
SNI (P10 or 60)	DRG [IBA1, ATF3, NF200, CGRP, IB4]	7 days	(191)
SNI (P10 or 56)	PWT PWL SC/DRG [IBA1, NF200, GFAP, IB4], WB	3, 7, 14, 21, 28, 38, 44 days 3, 7, 14, 21, 35, 51 days 7, 30 days	(192)
SNI (P10 or 33)	PWT, PWL, Cold pain behavior, Electrophysiology, IC SC [IBA1, BDNF, GFAP]	1, 7, 14, 21, 28, ±35 days 7, ±21 days	(211)
SNI (P3, 10, 21, 33, or 60)	PWT	0, 1, 7, 14, 28 days	(189)
CCI (P10 or 60)	PWT	0, 7, 14, 28 days	(189)
SNI (P14, 28, or 112)	PWT	0, 1 days, weekly up to 14 weeks	(190)

*P, post-natal day; CFA, complete Freud's' adjuvant; CAR, carrageenan; RNP; repetitive needle prick; SNI, spared nerve injury; CCI, chronic constriction injury; PWT, paw withdrawal threshold; PWL, paw withdrawal latency; IC, incapacitance meter; SC, spinal cord; DRG, dorsal root ganglion; IBA1, immunostaining for ionized calcium-binding adapter molecule 1 (microglia marker); c-Fos, proto-oncogene; CGRP, calcitonin gene-related peptide; ATF3, Activating transcription factor 3; IB4, isolectin B4; NF200, Neurofilaments protein; GFAP, glial fibrillary acidic protein; BDNF, Brain-derived neurotrophic factor; WB, western blot; ACTH, adrenocorticotropic hormone; EPM, elevated plus maze; OFA, open field arena; MWM, Morris water maze; CPP, conditioned place preference; FST, forced swim test; EMG, electromyography; NOR, novel object recognition*.

Neonatal pain models (first-hit) have been superimposed with secondary (adult) painmodels (second-hit) to evaluate the effects of early life pain on subsequent pain later in life. Like one-hit neonatal pain models, two-hit models are primarily induced in the rat. Subsequent pain typically occurs between P25 and P60 and varies from inflammatory (e.g., CFA, CAR) to tissue-breaking (e.g., RNP, incision) in nature (Table 4). These time periods in the rat most likely resemble humans classified as young adults and middle-aged adults, respectively. Alone these models are used to evaluate the effects of early life pain on later acute pain (215–217). Two-hit models provide evidence that single or repetitive noxious stimulation during the early post-natal period can affect subsequent acute pain later in life in regards to mechanical and thermal sensitivity (37, 206), learning and memory (61, 200), and microglial activation (16, 163). However, the impact that early post-natal injury has on chronically painful conditions during adulthood has not been evaluated in animal models.

**Table 4:** Rodent models of neonatal pain with subsequent pain and time of assessments.

**“2-Hit” Models**						
**1st Stimulus (age)**	**Basal outcome measure(s)**	**Age/Time**	**2nd Stimulus (age)**	**Outcome measure(s)**	**Age/Time**	**References**
**INFLAMMATORY** **+** **INFLAMMATORY PAIN**						
CFA (P0 or 14)			Capsaicin (P56–84)	Pain behavior PWT PWL Paw diameter SC [c-Fos]	5 min 5, 15, 30, 45, 60 min 5, 15, 30, 45 min 60 min	(195)
CFA (P1 or 14)			CFA (P84)	PWL	P85	(193)
CFA (PO)	PWT, PWL	P56	CFA (P56)	PWT, PWL	P57	(194)
CFA (P0, 1, 3, or 14)	PWL	P56–70	CFA (P56–70)	PWL	24 h	(134)
CFA (P0, 1, 3, or 14)	PWL	P56–70	Formalin (P56–70)	Pain behavior	Every 5 min for 60 min	(134)
CAR (P0, 1, 3, 5, 8, 10, 12, or 14)	PWT, PWL	P40	CFA (P40)	PWT, PWL	P41	(135)
CAR (P3)			CFA (P6, 13, 24, 40, or 62)	PWT	24 h	(135)
CAR (P3)	PWT, PWL	P120–125	CFA (P120–125)	PWT, PWL	24 h	(135)
CAR (P0 or P14)	PWL, Pain behavior	3, 6, 12, 18, 24, 48, 60 h, and P60	CFA (P61)	PWT, PWL, paw diameter	P62	(198)
CAR (P0)	PWL, tail flick	P40, 60	CFA (P60)	PWL, paw diameter	1, 7, 14, 21	(196)
CAR (P0, 8, or 14)	PWL, paw diameter	P40, 60	CFA (P60)	PWL	P61	(197)
CAR (P3)	PWL	P48, 49	CFA (P50)	PWL, tail flick, electrophysiology	24 h	(145)
CAR (P3 or 14)			Mustard oil (Colon) (P60–70)	PWL, visceromotor response	0, 30, 90, 150, 210 min	(199)
Formalin (P1–2 or 7–8)	PWL	P25	Picric acid + maternal deprivation (P25)	Pain behavior PWL	1–20 min P26	(96)
Formalin (P7–8)			Formalin (P25)	EPM, FST, MWM, nociceptive activity	P25–38	(200)
**INFLAMMATORY PAIN** **+** **STRESS**						
CAR (P0)			Chronic stress (P82–109)	MWM	P94–117, 144–162, 424–442	(61)
CAR (PO)			Chronic stress (P85–P105)	FST	7 days	(210)
**INFLAMMATORY** **+** **TISSUE-BREAKING PAIN**						
CFA (P1)	PWT	P35, 42, 49	Incision (P50)	PWT	4 h, 1, 2, 3, 4, 7 d	(171)
**INFLAMMATORY** **+** **NERVE INJURY**						
CFA (P0, 1, 3, or 14)			Sciatic nerve injection (P56–84)	SC [WGA-HRP density]	48 h	(134)
CFA (PO)	PWT, PWL	P56	SNL (P56)	PWT, PWL	P57, 60, 63, 70, 77, 84	(194)
CFA (tail) (P3)	PWT, PWL	P56	TNI (P56)	PWT, PWL	P57, 60, 63, 70, 77, 84	(194)
SNI (P10)	SC [IBA1]	6, 24, 72 h	LPS (P10–19, 19–21)	PWT	0, 30, 60, 90, 120, 150 min	(201)
**TISSUE-BREAKING** **+** **INFLAMMATION/OTHER PAIN**						
RNP (P0–7)			CFA (P56)	PWT	P57	(202)
RNP (P2–8)	PWL	P14–16, 35–36, 64–66	Formalin (P14–16, 35–37, or 64–66)	Pain behavior	2–7 days	(203)
**TISSUE-BREAKING** **+** **TISSUE-BREAKING PAIN**						
RNP (P0–7)	PWT PWL	1, 3, 5 h (P0–7), Weekly, P21–56 Weekly, P21–56	Incision (P56)	PWT SC [CGRP, VGLUT2]	1, 3, 4, 7, 9, 11 days	(37)
RNP (P0–7)	PWT	1, 3, 5 h (P0–7)	Incision (P42–56)	Electrophysiology	P42–56	(98)
RNP (P0–7)	PWT	1, 3, 5 h (P0–7), Weekly P21–56	Incision (P56)	PWT	1, 3, 5, 7, 9 days	(204)
RNP (P0–7)	PWT PWT, PWL	1, 3, 5 h (P0–7) Weekly P21–56	Incision (P56)	PWT, PWL	1, 3, 5, 7, 9 days	(214)
RNP (P2–14)			Acute stress (P15 or 20–21)	ACTH, corticosterone	5, 30, 60, or 90 min	(205)
Incision (P3, 6, 10, 21 or 40)	PWT	0, 4, 24, 48, 72 h	Incision (P17, 20, 24, 35, or 54)	PWT EMG	0, 4, 24, 48, 72 h, and 7 days 24 h	(100)
Incision (P3)			Incision (P56)	PWT, PWL, IBA1, EMG	0, 1, 3, 5, 7, 10, 14, 19, 21, 28 days	(16)
Incision (P3, 10, 17)			Incision (P90)	PWT, PWL, OFA, brain [c-Fos], novelty-induced hyponeophagia	0, 1, 2, 5 days	(97)
Incision (P3)	PWT	3, 4, 24 h	Incision (P42–49)	PWT, PWL EMG CPP, NOR	0, 1, 2, 4, 7, 9, 12, 17, 21 days 0, 24 h 24 h	(207)
Incision (P3)	PWT EMG SC [IBA1]	3, 4, 24, 48, 72 h 6 h 24 h	Incision (P42)	PWT, PWL EMG, OFA SC [IBA1]	0, 1, 2, 3, 7, 10, 14, 17, 21 days 24 h P6	(206)
Incision (P3)			Incision (P70)	PWT, PWL, inclined plate test SC [IBA1] Electrophysiology	0, 1, 3, 5, 7, 14, 21 days 0, 1, 3, 5 7 days 7 days	(163)
**OTHER PAIN** **+** **OTHER PAIN**						
Orogastric suctioning (P2–11)	PWL	P60	Colorectal distention (P60)	PWL, visceral sensitivity	24 h	(209)
Lesion to motor cortex (P7)			Lesion to motor cortex (P70)	SC histology	P70	(208)
Electrical stimulation (P10)			Electrical stimulation ± Capsaicin (adult; *not specified*)	PWT SC [IBA1]	0, 3, 24, 48 h 48 h	(164)

*P, post-natal day; CFA, complete Freud's' adjuvant; CAR, carrageenan; RNP; repetitive needle prick; SNI, spared nerve injury; CCI, chronic constriction injury; PWT, paw withdrawal threshold; PWL, paw withdrawal latency; IC, incapacitance meter; SC, spinal cord; DRG, dorsal root ganglion; IBA1, immunostaining for ionized calcium-binding adapter molecule 1 (microglia marker); c-Fos, proto-oncogene; CGRP, calcitonin gene-related peptide; ATF3, Activating transcription factor 3; IB4, isolectin B4; NF200, Neurofilaments protein; GFAP, glial fibrillary acidic protein; BDNF, Brain-derived neurotrophic factor; WB, western blot; ACTH, adrenocorticotropic hormone; EPM, elevated plus maze; OFA, open field arena; MWM, Morris water maze; CPP, conditioned place preference; FST, forced swim test; EMG, electromyography; NOR, novel object recognition*.

## Effects of Early Life Pain on Chronic Pain in the Adult

Chronic pain is a major global health concern affecting more than 1.5 billion people worldwide (218). Over the last decade, studies concerning the health status of adults with a history of varying degrees of prematurity, early life procedural pain, and/or early life surgery have been conducted to understand the effects of early life pain on diseases in adulthood (109, 219–221). Retrospective studies are very challenging to conduct because detailed medical records are not always accessible and/or there is a loss of patient retention due to geographic relocation or denied consent. These studies are further limited because of variability in details describing procedures performed including number of procedures, time of occurrence, and dose/route of drug administration in addition to differences in physician's and/or health facility's standard protocols, or lack thereof. Finally, early life painful events may not be captured during childhood or adult examinations because there is lack of patient recall, medical exam time constraints, or focused questions to gather pertinent information to current condition.

Regardless, untreated early life pain has been suggested to be a significant contributor to the development of chronic pain in children and adults (17, 97). It is speculated that this notion would be supported by animal models. However, two-hit models have focused on the effects of early pain on secondary acute pain in adult rats as previously discussed. Whether noxious stimuli during the neonatal period predisposes a patient (human or animal) to pain-associated with chronic painful conditions remains unknown. There are multiple animal models of chronic pain (222, 223) that could be coupled with an early life pain model to investigate whether there is an impact on chronic pain conditions. Well-documented chronic pain rodent models can be used to provide foundational data on pain responses and mechanistic pathways, and early life pain could be superimposed on this model to evaluate the influence of early life pain on chronic pain states. Indeed, this approach has been communicated in abstract form where we found that neonatal RNP pain appears to heighten reflexive and complex pain behaviors following chemical induction of osteoarthritis in adult rats, compared to osteoarthritis alone (224). However, there may be opportunities where using large animal models (e.g., dogs, pigs) would be preferred as these models are preexisting and better mimic human disease (225–228).

To date there is a complete lack of published clinical or basic science information on the effects of early life pain on persistently painful conditions in adults. Assessing ‘naturally occurring,’ or spontaneous, chronic pain conditions in animals with a history of early life pain could add to our understanding of the consequences of early life pain on later chronic pain (229). Companion animals commonly are surgically de-sexed, or sterilized (e.g., ovariohysterectomy or orchidectomy), at an early age (<6 months old), providing a natural model of early life surgery and pain. Such studies would be best performed prospectively, although one obvious shortcoming of such studies would be the removal of the influence (positive or negative) of sex hormones. To avoid hormonal confounders and the risk of missing the “critical period” of neurodevelopment other elective canine procedures may be used, such as tail docking (3–7 days old) or ear cropping (7–12 weeks old). Another clinically relevant example would be studying piglets who undergo painful procedures as a part of routine management practices (e.g., tail docking, castration, ear notching). Pigs commonly develop joint pain later in life and relevant outcome measures have been described. Interestingly, a piglet model has been developed to study the impact of NICU procedures (e.g., mechanical ventilation) in preterm infants (230), which could easily become a two-hit model.

## Conclusions and Future Directions

Despite significant basic science work, chronic pain remains a significant public health crisis, where the prevalence of new cases has persistently increased in both adolescents (231) and adults (232–234). The influence of predisposing risk factors, such as age, gender, and obesity on chronic pain have been investigated for several decades, but there has been limited exploration of additional underlying risks, such as early life pain.

A growing body of evidence reports long-term consequences of early life pain in humans, and elegant animal studies are gradually shedding light on the neuroanatomical and functional mechanisms responsible for this (18, 88, 119, 235, 236). However, there is also the distinct possibility that early life pain may impact the degree and severity of chronically painful conditions that occur much later in life. To investigate this relationship, a series of systematic studies need to be conducted. First, a suitable two-hit model incorporating a painful procedure that consistently happens during the neonatal period and a disease that causes chronic pain in adulthood is needed. Second, an appropriate battery of outcome measures that are clinically relevant should be explored to evaluate changes to neurophysiology, psychology, and behavior. Finally, mechanisms responsible for long-term effects of early life pain should be determined using these models, and this will lay the foundation for the development of therapies to improve the quality of life of patients who suffer the consequences of early life pain experiences.

## Author Contributions

MW co-conceived the review, performed the literature searches and literature summaries, drafted and revised the manuscript, approved the final manuscript as submitted, and agreed to be accountable for all aspects of the work. BL co-conceived the review, assisted in the design of the content, made critical input and assisted in revisions, approved the final manuscript as submitted, and agreed to be accountable for all aspects of the work.

### Conflict of Interest

The authors declare that the research was conducted in the absence of any commercial or financial relationships that could be construed as a potential conflict of interest.
